# A six-chambered heart: cor triatriatum sinister with double-chambered right ventricle in association with ventricular septal defect

**DOI:** 10.1186/s43044-022-00246-0

**Published:** 2022-02-16

**Authors:** Vivek Jaswal, Pratyaksha Rana, Vidur Bansal, Krishna Prasad Gourav, Arun Sharma, Shyam Kumar Singh Thingnam

**Affiliations:** 1 Department of Cardiothoracic and Vascular Surgery, Post Graduate Institute of Medical Education and Research, Chandigarh, 160012 India; 2 Department of Radiodiagnosis and Imaging, Post Graduate Institute of Medical Education and Research, Chandigarh, India; 3 Department of Anesthesia, Post Graduate Institute of Medical Education and Research, Chandigarh, India

**Keywords:** Cor triatriatum, Double, Right ventricle, Left vena cava

## Abstract

**Background:**

Cor triatriatum has been described as a heart with three atria in which the left atrium (cor triatriatum sinistrum) or right atrium (cor triatriatum dextrum) is divided into two compartments by a fold of tissue, a membrane, or a fibromuscular band. Double-chambered right ventricle, on the other hand, is identified by the presence of an anomalous muscle bundle dividing the right ventricle into two chambers.

**Case presentation:**

Here, we describe the case of a child who had a combination of both of these rare entities, effectively creating a heart with six chambers. The child underwent a successful intracardiac repair.

**Conclusions:**

The association of CTS with DCRV forming a “6-chambered heart” is extremely rare. Awareness of its existence and accurate preoperative diagnosis has important implications in its surgical repair with all the components of this disease spectrum, further increasing the complexity of a successful surgical repair.

## Background

In cor triatriatum sinister (CTS), the proximal chamber of the left atrium (LA) receives venous blood, whereas the distal chamber is in contact with the atrioventricular valve and contains the atrial appendage and the true atrial septum bearing the fossa ovalis. The membrane that separates the atrium into two parts varies significantly in size and shape. In double-chambered right ventricle (DCRV), an anomalous muscle band divides the right ventricular cavity into a proximal and a distal chamber [1]. In the pediatric population, CTS may be associated with major congenital cardiac lesions but its association with DCRV has not been described thus far in the literature.

## Case presentation

A 2-year-old child is presented with the complaints of shortness of breath while playing and moderate cyanosis for the past one and a half years. The symptoms had progressed over the past 3 months. On examination, the child had an oxygen saturation of 80% on room air and a systolic murmur in the pulmonary area.

**Fig. 1:**
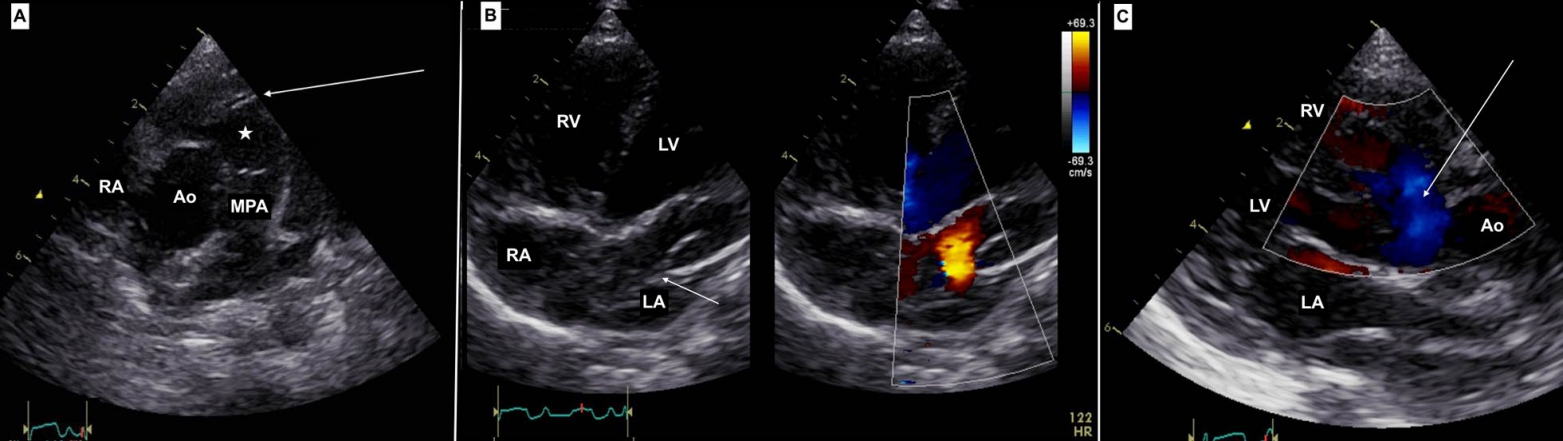
Transthoracic echocardiography. **A** Parasternal short axis view showing the hypertrophied muscle bands (arrow) with distal infundibular chamber (*). **B** Apical four-chamber view showing the membranous partition in the LA (arrow) with turbulent flow across its aperture. **C** Parasternal long axis view showing the perimembranous VSD (arrow). (Ao-aorta)

**Fig. 2:**
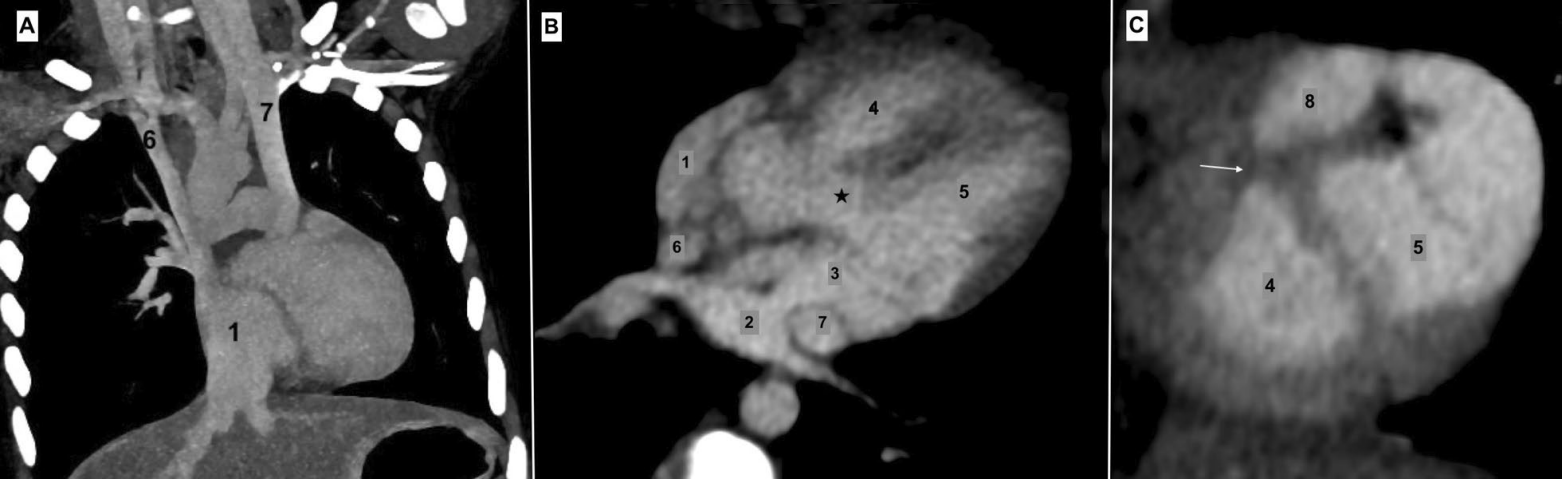
CT Angiography. **A** Coronal section showing double SVC draining into RA. **B** Four-chamber view showing cor triatriatum, VSD (*) and double SVC. **C** Two-chamber view showing the infundibular chamber and the hypertrophied obstructing muscle bands (arrow) dividing the RV into two chambers. 1—Right atrium, 2—posterior chamber of left atrium, 3—anterior chamber of left atrium, 4—right ventricle, 5—left ventricle, 6—right superior vena cava, 7—left superior vena cava, 8—infundibular chamber

**Fig. 3:**
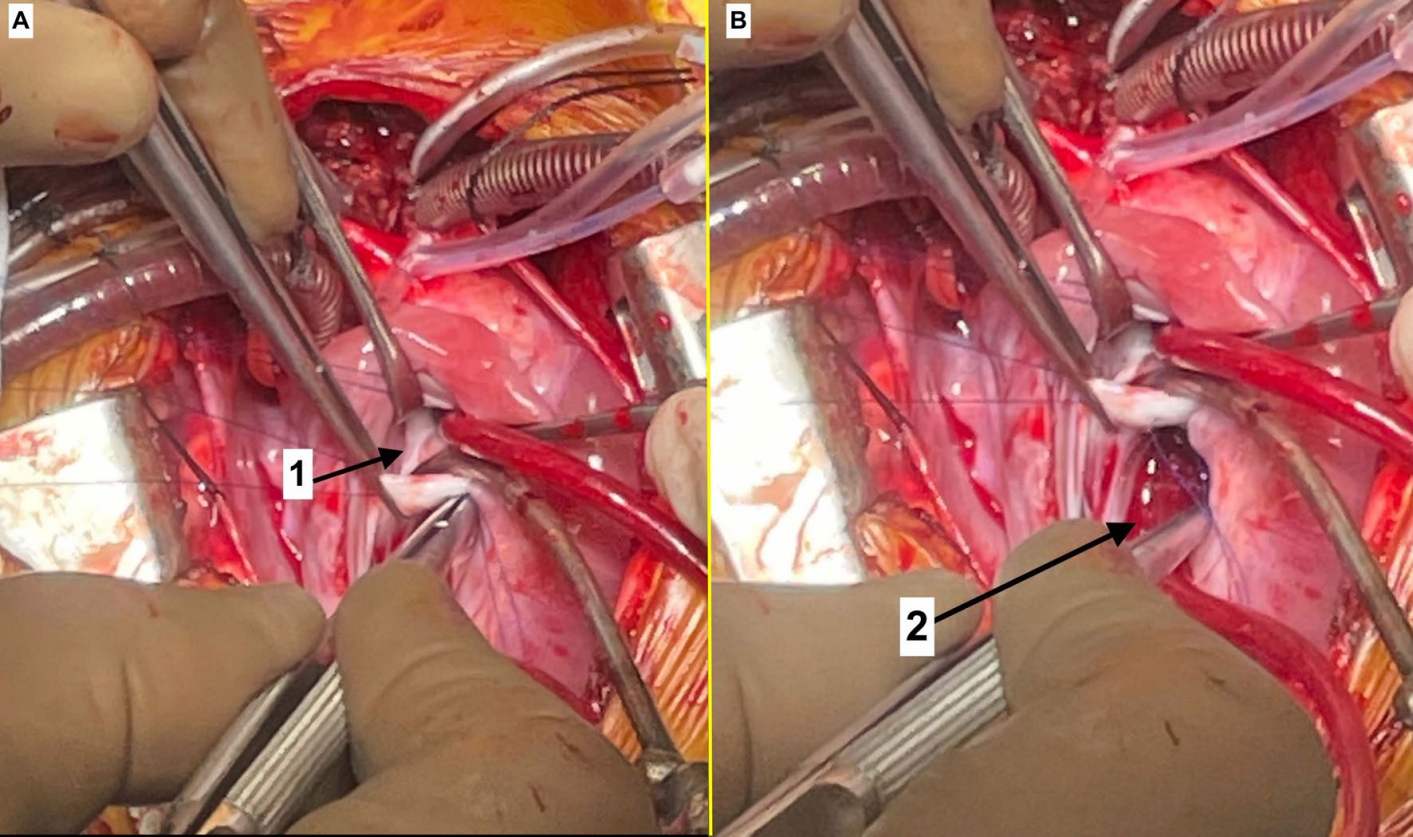
Intra-operative images. **A** The membranous partition (1) of LA as seen through the PFO. **B** The dilated coronary sinus (2)

The child was further evaluated with basic investigations and a transthoracic echocardiography, which revealed CTS in association with DCRV, having a gradient of 70 mmHg across it, a perimembranous ventricular septal defect (VSD) in the proximal chamber of right ventricle, a small atrial septal defect (ASD) between the right atrium (RA) and distal chamber of the LA in the septum secundum and persistent left superior vena cava (PLSVC) (Fig. 1). The atrial membrane that separated the proximal and distal chambers of LA, was mildly restrictive with a mean gradient of 4 mmHg across it. The coronary sinus was dilated. Computed tomography (CT) angiography confirmed the above findings (Fig. 2). The child was then planned for a definitive intracardiac repair. After a median sternotomy, the surface anatomy of the heart was assessed. Invasive pressure monitoring showed RV systolic pressures (89 mmHg) almost equal to the systemic systolic pressure (91 mmHg). Cardiopulmonary bypass (CPB) was instituted with cannulation of the ascending aorta, right superior vena cava (RSVC), LSVC and inferior vena cava (IVC). The heart was arrested in diastole with Del Nido cardioplegia. Patient was cooled to 34 °C and the RA was opened. Through the ASD, the distal chamber of the LA in contact with the mitral valve was entered. A small opening between the proximal and distal chambers of the LA was visualized. The membranous partition (Fig. 3A) adjoining this small opening was excised taking care not to injure the wall of the dilated coronary sinus (DCS) (Fig. 3B) and the underlying mitral valve. The fibrotic os infundibulum and hypertrophied obstructing parietal and septal muscle bands were excised facilitating an Hegar’s dilator that was appropriately sized according to the Rowlatt chart. The VSD and ASD were closed using an autologous 0.6% glutaraldehyde treated pericardial patch. The child was successfully weaned off CPB. Invasive pressure monitoring showed RV pressures of 33/1 mmHg and LV pressures of 75/30 mmHg. Thus, postoperatively, the RV systolic pressures were reduced to 40% of the systemic systolic pressures. The child was shifted to the ICU on ionotropic support. The child was extubated on first postoperative day but subsequently developed superficial surgical site infection. The child was discharged on 12th postoperative day and is currently doing well in follow-up.

## Discussion

The incidence of cor triatriatum has been variously reported as 0.1–0.4% [2]. Patients with CTS are present with symptoms similar to mitral stenosis. These patients usually have pulmonary hypertension and signs of RV failure [3]. PLSVC is present in 0.5% of the general population and results from failure of occlusion of the left cardinal vein in embryonic life [4]. It drains into the right atrium through the coronary sinus in most cases and a DCS on imaging should raise suspicion of its presence. A DCS can have implications in repair of such a lesion as the inter-atrial septum can only be excised to a limited extent to gain entry into the left atrium. Further, a DCS can complicate excision of the membranous partition in the LA as the roof of a DCS can be mistaken for the LA membrane.

A VSD may communicate with either the proximal or distal chamber, leading to a greater shunt in the latter situation. Development of RV outflow tract obstruction occurs in 3–7% of patients with membranous VSD’s within the first year of life [5, 6].

CTS is amenable to surgical correction with excellent results when diagnosed. Long-standing inflow obstruction to the left ventricle leads to pulmonary venous and arterial hypertension along with right ventricular dysfunction. Additional presence of DCRV along with CTS adds to the complexity as it leads to further RV hypertrophy, exposes the RV to pressure overload and increases the predisposition to early RV dysfunction. Hence, the need for early surgical intervention in such cases. In our case, the six chambers of the heart were correctly diagnosed by preoperative echocardiography and CT-angiogram, facilitating the surgical planning beforehand. A six-chambered heart as described here was described around 50 years back but the morphology was entirely different [7].

## Conclusions

The association of CTS with DCRV forming a “6-chambered heart” is extremely rare. Awareness of its existence and accurate preoperative diagnosis has important implications in its surgical repair with all the components of this disease spectrum further increasing the complexity of successful surgical repair.

## Data Availability

Yes.

## Abbreviations

ASD: Atrial septal defect
CTS: Cor triatriatum sinistrum
CPB: Cardiopulmonary bypass
DCRV: Double-chambered right ventricle
DCS: Dilated coronary sinus
LA: Left atrium
LV: Left ventricle
PLSVC: Persistent left superior vena cava
RA: Right atrium
RV: Right ventricle
VSD: Ventricular septal defect
